# Biomechanical knee energy harvester: Design optimization and testing

**DOI:** 10.3389/frobt.2022.998248

**Published:** 2022-10-05

**Authors:** Moran Gad, Ben Lev-Ari, Amir Shapiro, Coral Ben-David, Raziel Riemer

**Affiliations:** ^1^ Mechanical Engineering Department, Ben-Gurion University of the Negev, Beer-Sheva, Israel; ^2^ Industrial Engineering and Management Department of Ben-Gurion University of the Negev, Beer-Sheva, Israel

**Keywords:** biomechanical knee energy harvester, exoskeleton, reduced effort, simulation, design optimization

## Abstract

Biomechanical energy harvesters are designed to generate electrical energy from human locomotion (e.g., walking) with minimal or no additional effort by the users. These harvesters aim to carry out the work of the muscles during phases in locomotion where the muscles are acting as brakes. Currently, many harvesters focus on the knee joint during late swing, which is only one of three phases available during the gait cycle. For the device to be successful, there is a need to consider design components such as the motor/generator and the gear ratio. These components influence the amount of electrical energy that could be harvested, metabolic power during harvesting, and more. These various components make it challenging to achieve the optimal design. This paper presents a design of a knee harvester with a direct drive that enables harvesting both in flexion and extension using optimization. Subsequently, two knee devices were built and tested using five different harvesting levels. Results show that the 30% level was the best, harvesting approximately 5 W of electricity and redacting 8 W of metabolic energy compared to walking with the device as a dead weight. Evaluation of the models used in the optimization showed a good match to the system model but less for the metabolic power model. These results could pave the way for an energy harvester that could utilize more of the negative joint power during the gait cycle while reducing metabolic effort.

## 1 Introduction

Biomechanical energy harvesters are exoskeletons designed to generate electrical energy from human locomotion (e.g., walking) and provide an alternative to batteries as an electrical power source for portable electronics (GPS, laptops, etc.). Many current harvesting devices aim to carry out part of the work of the muscles during phases in human locomotion where the muscles are acting as brakes (i.e., negative muscle work) (Donelan et al., 2008), (Niu et al., 2004), see Figure 2. This leads to regenerative braking, which generates electrical energy in a similar way to a hybrid car and may reduce the user’s effort. For energy-harvesting devices to be useful, it is important that the addition of effort (metabolic rate) required to carry the device on the body and to generate electrical energy is minimal or negative (Schertzer and Riemer, 2015).

While the exact conditions required for achieving harvesting while reducing metabolic energy are unknown, there where evidence in the past that this is possible (Donelan et al., 2008), (Shepertycky and Li, 2015). Further, a recently developed energy harvester utilized late swing to produce 0.25 W of electrical energy while reducing the metabolic energy (relative to walking with no device) by 6 W (Shepertycky et al., 2021). There could be several types of biomechanical energy; the main ones are a backpack that uses the oscillation of the center of mass, a shoe base that uses sole compression to generate electrical energy, and a joint base that uses the relative motion between the segments (Riemer and Shapiro, 2011). Currently, the most successful harvesters are devices that target negative muscle work performed at the knee, as this is a joint with a relatively large amount of negative work performed at that location (Figure 2). However, current knee devices [(Donelan et al., 2008), (Shepertycky and Li, 2015), (Shepertycky et al., 2021)] are focused on the late swing (end on K4), which means that they can only make use of less than half of the potential energy that could be harvested during a full gait cycle (Riemer and Shapiro, 2011). Moreover, the ability of these devices in harvesting is limited, due to their mechanical design, which uses a roller clutch (Donelan et al., 2008) or a combination of a roller clutch and cable (Shepertycky and Li, 2015; Shepertycky et al., 2021). In this context, they can harvest only at the flexion of the knee.

During device design, several parameters must be considered. First, the device must be attached to the body, and carrying its mass requires additional metabolic power from the user. The metabolic rate required to carry the device is based on the user’s walking speed and the device’s mass and location (Browning et al., 2007)– (Schertzer and Riemer, 2014) (Stuempfle et al., 2004; Soule and Goldman, 1969). Other important design components and their specifications include the motor/generator and the gear ratio. (Note that since the electrical motor is also used as the generator in this study, we use these terms interchangeably). All the aforementioned components have an effect on the other components as well as on the overall performance. Further, some of the design parameters improve the device in one domain, but make it worse in another. For example, the combination of gear, motor, and current determine the resistance torque of the device; a higher gear ratio may produce more electricity, but it might lower the conversion efficiency of the system due to increased friction (higher gear ratios are less efficient). Further, a higher gear ratio will increase the inertia and require more effort from users, especially in non-harvesting phases of the gait. Harvesting more electrical energy will increase the applied torque and forces working on the system, which affects the stress on the structure, its dimensions and mass. All of the above make the task of finding the optimal design challenging.

To address this issue, Li et al. (2009) used a static model (i.e., one that did not include system dynamics) to optimize device efficiency as a function of the gear ratio and the external electrical load of the motor. However, they found that dynamic effects can account for as much as half of the resistance torque applied by the device, which means that the device parameters chosen by their optimization might not have been optimal. Martin et al. (2016) proposed a model that included dynamic effects for a lower limb-driven energy harvester. However, they did not suggest a framework for optimizing the design parameters of the harvester.

The aim of this study is to design and build a knee energy-harvesting device that could generate electricity during joint mechanical negative work phases of the gait, both at flexion and extension. To achieve the best design, we used an optimization process that considers the design parameters of our device, such as the generator’s electrical specification, mass, and gear ratio. The optimization framework was based on a dynamic model of the electromechanical system. Further, we were able to control the torque applied to the knee by the device using technology from our previous study (Cervera et al., 2016). The optimization objective is to minimize the total cost of harvesting (TCOH), which consists of the change in metabolic power when walking with the device vs. without it divided by the electrical power produced (Shepertycky and Li, 2015).

## 2 Methods

### 2.1 Overview

The design of the device entailed the following steps

1) Conceptual design of the system,
2) Optimization to minimize the total cost of harvesting, and
3) Calculation of the TCOH using three models. The models were a)
  a) Model for the electrical power generated during the system’s operation
  b) A dynamic model of the electromechanical system, including prediction of the device’s applied torque profile; and
  c) A prediction model for the change in the metabolic power as a function of the device’s mass, location on the body, and torque.

### 2.2 Conceptual design of device

**FIGURE 1:**
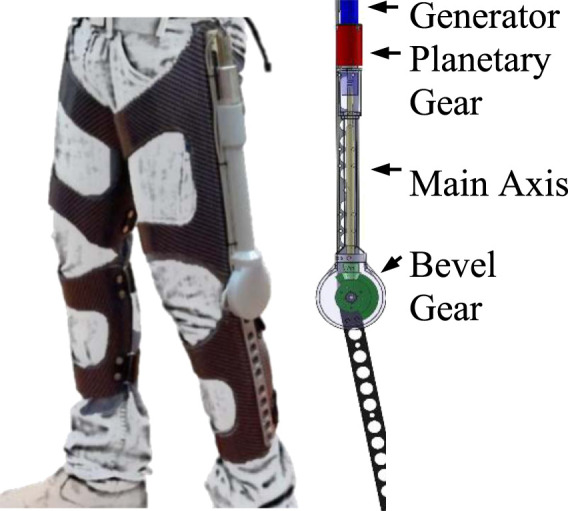
General mechanical design of the harvesting device mounted on the knee brace.

**FIGURE 2:**
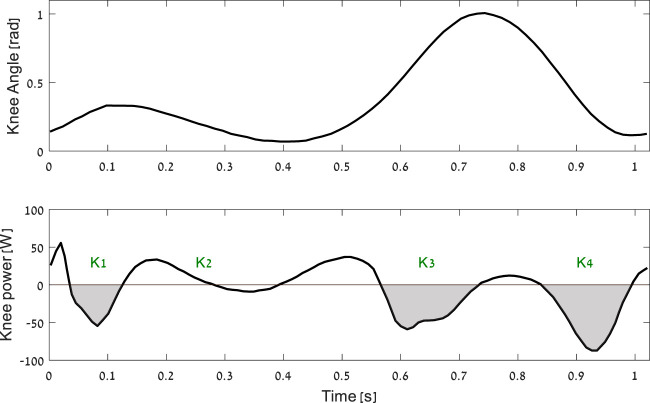
The mechanics of the knee joint during a walking gait cycle from heel strike to heel strike. The shaded areas (i.e., K1, K3, and K4) are the phases of negative net muscle work at joint level [based on (Winter, 2009)].

The device is based on a gear train that increases the angular velocity of the knee to rotate a brushless DC motor (functioning as an electrical generator). The apparatus of the harvesting device is mounted on an orthopedic knee brace (Figure 1). Using a micro-controller, the device controls the torque applied to the knee, which in turn controls the amount of energy to be harvested (Cervera et al., 2016). To reduce the metabolic cost of carrying the device, the gear and generator are located as high as possible on the thigh. Energy harvesting occurs during negative joint work periods, specifically during phases K1, K3, and K4 of the gait cycle (Figure 2). To enable harvesting during both knee flexion and extension, the motor is connected with no clutch mechanism (direct drive). We also tested an option for two oppositely oriented roller clutches that could enable harvesting in both directions, but similar to (Donelan et al., 2008), we found that this design was too heavy.

### 2.3 Objective function

The objective function is to minimize the total cost of harvesting (TCOH, where the optimization parameters are the generator model (from a list of possible generators), the gear ratio, and the torque/current profile. The general optimization formula is

$$\begin{array}{c} \min TCOH\left(v\right)\\ {\text{subject}\;\text{to}:c}_{k}\left(v\right)\geq 0\;for\;k\in \left[0,I\right] \end{array}$$ (1)

where $c_{k}\left(v\right)\geq 0$ and k∈I are inequality constraints representing the physical limitations of the electromechanical system and the user’s ability, respectively, and ν is a vector of optimization variables (i.e., torque/current profile, gear ratio, and motor model). The TCOH is defined as

$$TCOH=\frac{\Delta P_{met}}{P_{eff}}$$ (2)

where $\Delta P_{met}$ is the estimated additional metabolic rate for using the harvester relative to the metabolic rate during walking without the device, defined in (14), and $P_{eff}$ is the effective harvested electric power, defined in (3).

### 2.4 Models

This section describes the mathematical derivation of the three models: 1) system power model, 2) system torque model, and 3) change in metabolic rate due to carrying the device and change in the net joint work [based on (Margaria, 1968)].

#### 2.4.1 System’s electrical power model

The power model estimates the effective electrical power that the harvester will be able to produce. We define the system’s effective power as

$$P_{eff}=P_{gen}-P_{lost}$$ (3)

where $P_{gen}$ is the total electrical power produced by the generator, and $P_{lost}$ is the total power lost during the system’s energy conversion. The total power produced by the generator, $P_{gen}$ , is given by

$$P_{gen}=E_{g}i_{g}$$ (4)

where $i_{g}$ is the equivalent DC current that flows through the generator, which is controlled by a closed-loop circuit proportional–integral–derivative (PID) controller, and $E_{g}$ is the generator electromotive force (EMF). In our models, we used the single-phase DC equivalent of our generator constants. Thus, the generator EMF is given by

$$E_{g}=\left(\omega_{k}G.R\right)K_{e}$$ (5)

where $\omega_{k}$ is the angular velocity of the knee, and G.R is the gear ratio of the system. Multiplying $\omega_{k}$ by G.R results in the generator’s angular velocity. $K_{e}$ is the generator speed constant. The total power lost due to conversion, $P_{lost}$ , is

$$P_{lost}=2i_{g}E_{d}+i_{g}^{2}R_{i}$$ (6)

where $E_{d}$ is the reverse current voltage that is lost on the three-phase diode bridge (where two of the diodes conduct at any given timepoint), and $R_{i}$ is the DC equivalent internal resistance. To use these equations with a brushless direct current (BLDC) generator, the parameters from the specification data sheet need to be converted to single-phase DC equivalent constants (see Appendix A).

#### 2.4.2 System’s torque model

This model calculates the torque applied by the harvesting device to the knee during the negative joint mechanical power phase of walking, which is formulated as

$$T_{sys}=T_{g}+T_{f}-T_{I}$$ (7)

where $T_{sys}$ is the torque applied by the device to the knee, $T_{I}$ is the torque due to the angular acceleration and moment of inertia (MOI) of the system, $T_{f}$ is the friction torque, and $T_{g}$ is the contribution of the generator to the total applied torque at the knee. $T_{g}$ is given by

$$T_{g}=\left(i_{g}K_{m}\right)G.R$$ (8)

where $K_{m}$ is the generator’s DC equivalent torque constant. $T_{I}$ is given by

$$T_{I}=I_{sys}\alpha_{k}$$ (9)

where $\alpha_{k}$ is the equivalent angular acceleration, and $I_{sys}$ is the equivalent of the MOI of the harvester, as experienced at the user’s knee level. It is comprised of the MOI due to different parts of the harvester, where each part experiences different angular acceleration. Hence, $I_{sys}$ is given by

$$\begin{array}{c} I_{sys}=I_{g^{@knee}}+I_{a^{@knee}}+I_{b}\\ I_{sys}=I_{g^{@knee}}+I_{a^{@knee}}+I_{b} \end{array}$$ (10)

where $I_{g}$ is the MOI of the generator, which is driven by the total gear ratio, $G.R_{tot}$ ; this includes the planetary gear ratio and the bevel gear ratio. $I_{a}$ is the sum of the MOI of the main axis and the planetary gear, which is driven by the gear ratio $G.R_{b}$ , that is, the bevel gear ratio. Finally, $I_{b}$ is the MOI of the knee brace and the harvester structural base. $T_{f}$ is the friction torque due to the gear trains and bearings. Both the bevel gear and the planetary gear cause a viscous friction, which is modeled as a single viscous friction factor. The various bearings cause an additional constant friction torque, which is modeled as a constant friction factor. Thus,

$$T_{f}=\left(C_{1}{\dot{\theta }}_{k}+C_{2}\right)sign\left({\dot{\theta }}_{k}\right)$$ (11)

where $C_{1}$ is the viscous friction coefficient, ${\dot{\theta }}_{k}$ is the angular velocity at knee level, $C_{2}$ is the constant friction coefficient, and the sign function determines the direction of the torque based on the angular velocity. However, none of the manufacturers of gears provide the above coefficients. Thus, we modified our torque model, (7), as follows

$$T_{sys}=\frac{T_{g}-T_{I}}{\eta }$$ (12)

where η is the system efficiency rate, which is mainly a function of the gear ratio. To determine the system efficiency, we used the vendor’s specification document where efficiency is defined as a function of gear ratio. Note that the bevel gear and the planetary gear were chosen from among the options provided by the vendor so that the dimensions and weights would be reasonable for a wearable device.

#### 2.4.3 Estimation of user’s additional metabolic power

$\Delta P_{met}$ is the estimated additional metabolic consumption of the user when wearing the harvester in comparison to his/her metabolic rate when walking without the device. Hence,

$$\Delta P_{met}=P_{met}^{harvesting}-{P_{met}^{No\_device}}_{\cdot }$$ (13)

The model for $\Delta P_{met}$ is given as

$$\Delta P_{met}=4.0P_{mech}^{POS}+0.8P_{mech}^{NEG}+\Delta P_{mass}$$ (14)

where $P_{mech}^{POS}$ is the mechanical power that the device requires from the user during the positive phases, which is multiplied by four to estimate the change in metabolic rate. $P_{mech}^{NEG}$ is the mechanical power (has negative values) that the device provides at the joint level, thus reducing muscular effort.

$\Delta P_{mass}$ is the power that the user expends as a result of carrying the harvester due to its weight and location on the body. The coefficients for converting mechanical work to metabolic rate in (14) were based on (Margaria, 1968).

To model the effect of carrying the mass on the body, we used equations from (Schertzer and Riemer, 2015), which resulted in

$$\Delta P_{mass}\left(dm,S,L\right)=P_{mass}\left(dm,S,L\right)-P_{mass}\left(0,S,L\right)$$ (15)

where $\Delta P_{mass}$ is the difference in the metabolic rate for a given location of the additional mass on the body, dm is the device mass, S is the walking speed, and L is the location of the device mass on the body. In the optimization, the different combinations of motor and gear only affect the device mass and the location of the center of mass on the thigh. To calculate the difference in the metabolic rate of walking with and without the exoskeleton mass at a given speed of 1.3 m/s, the following metabolic rate prediction equations from (Schertzer and Riemer, 2015) were used:

$$P_{mass}^{back}=\exp\left(0.51+0.22S+0.011dm\right)$$ (16)

$$P_{mass}^{knee}=\exp\left(0.59+0.206S+0.059dm\right)$$ (17)

where $P_{mass}^{back}$ is the metabolic rate for carrying a mass on the back (at waist level), and $P_{mass}^{knee}$ is the corresponding metabolic rate for the knee. As can be seen, these equations apply to masses carried at the back/waist and knee levels; thus, interpolation was required to make them applicable to the thigh. The metabolic rate for wearing the device was calculated using a linear interpolation that takes into consideration the location of the center of mass between the knee and the back (waist).

Finally, to calculate $P_{mech}$ (14), the mechanical power (either negative or positive) that the user either expends or receives due to the harvester mechanism, the following equation is used:

$$P_{mech}=\left(T_{I}+T_{f}\right)\omega_{k}-T_{gen}\omega_{k}$$ (18)

where $T_{I}$ is the torque caused by the inertia and angular acceleration, as given in (9); $T_{f}$ is the friction torque, as given in (11); and $T_{gen}$ is the generator active torque, as given in (8).

#### 2.4.4 Optimization formulation and constraints

The goal of the optimization is to minimize the TCOH. Thus, the optimization problem, given in (2) and repeated here for convenience, is as follows:

$$\min TCOH=\frac{\Delta P_{met}}{P_{eff}}$$ (2)

subject to

$$Main\;Constraint:\;\{T_{n}^{sys}\leq bT_{n}^{human},0<b<1$$ (19)

$$Secondary\;Constraints:\;\left\{ \begin{array}{l} G.R<512\\ T^{sys}<15\left[Nm\right]\\ e_{sys}>3\left[V\right]\\ i_{sys}<10\left[A\right] \end{array}\right.$$ (20)

Where TCOH is the cost function. The optimization is bounded by the main constraint (19), where $T_{n}^{sys}$ is the total torque that the system applies to the knee at time sample n , given in (7), and $T_{n}^{human}$ is the average torque profile of a human for normal walking at time sample n of their gait cycle, as given in (Winter, 2009). *b* is a scaler that defines the ratio between the torque that the device applies on the user and the torque produced by the user when walking without the device. Although the optimal ratio (*b*) is unknown (Shepertycky et al., 2021), (Riemer and Shapiro, 2011), based on preliminary testing with earlier prototypes in our lab as well as the results of Collins et al. (2015) (who found that the optimal spring stiffness for their device was slightly less than half of the ankle stiffness), we defined the optimal ratio as b=0.5 .

The inequality constraints in (20) represent the physical limitations of the harvester system components. The upper bound of the gear ratio was selected based on the data sheet of the Apex planetary gearbox—AM032 series with three stages (Apex Dynamics, Long Island, NY, United States). These planetary gearboxes are suitable for our harvester since they enable the application of a maximum torque of 15 Nm at the knee, which is more than half of the maximum of the knee joint torque at the K3 and K4 phases. In addition, they have weights and sizes that are suitable for a wearable device. Note that we did not harvest at K1, as it requires a higher gear ratio than K3 and K4.

The optimization controls the harvester torque profile. This torque profile was designed based on our own electric controller (Cervera et al., 2016), (Rubinshtein et al., 2014) and on our experimental results for walking. $e_{sys}$ is the harvester voltage at the generator’s output, and it is bound by the maximum conversion ratio of the power stage $\left(A_{conv}^{\mathrm{MAX}}\right)$ and the maximum output voltage $\left(e_{C}^{\mathrm{MAX}}\right)$ . Thus, the minimum harvest voltage is given by

$$e_{sys}^{\mathrm{MIN}}=e_{C}^{\mathrm{MAX}}/A_{conv}^{\mathrm{MAX}}\cdot$$ (21)

It should be noted that if the storage capacitor is not full, it might still be possible to harvest, but this condition cannot be guaranteed. Therefore, in the optimization algorithm, we chose not to harvest energy when $e_{sys}$ was less than 3 V. The last constraint is $i_{sys}^{\mathrm{MAX}}$ , which is the maximum current that the converter is rated for $\left(i_{sys}^{\mathrm{MAX}}=10\right)$

**FIGURE 3:**
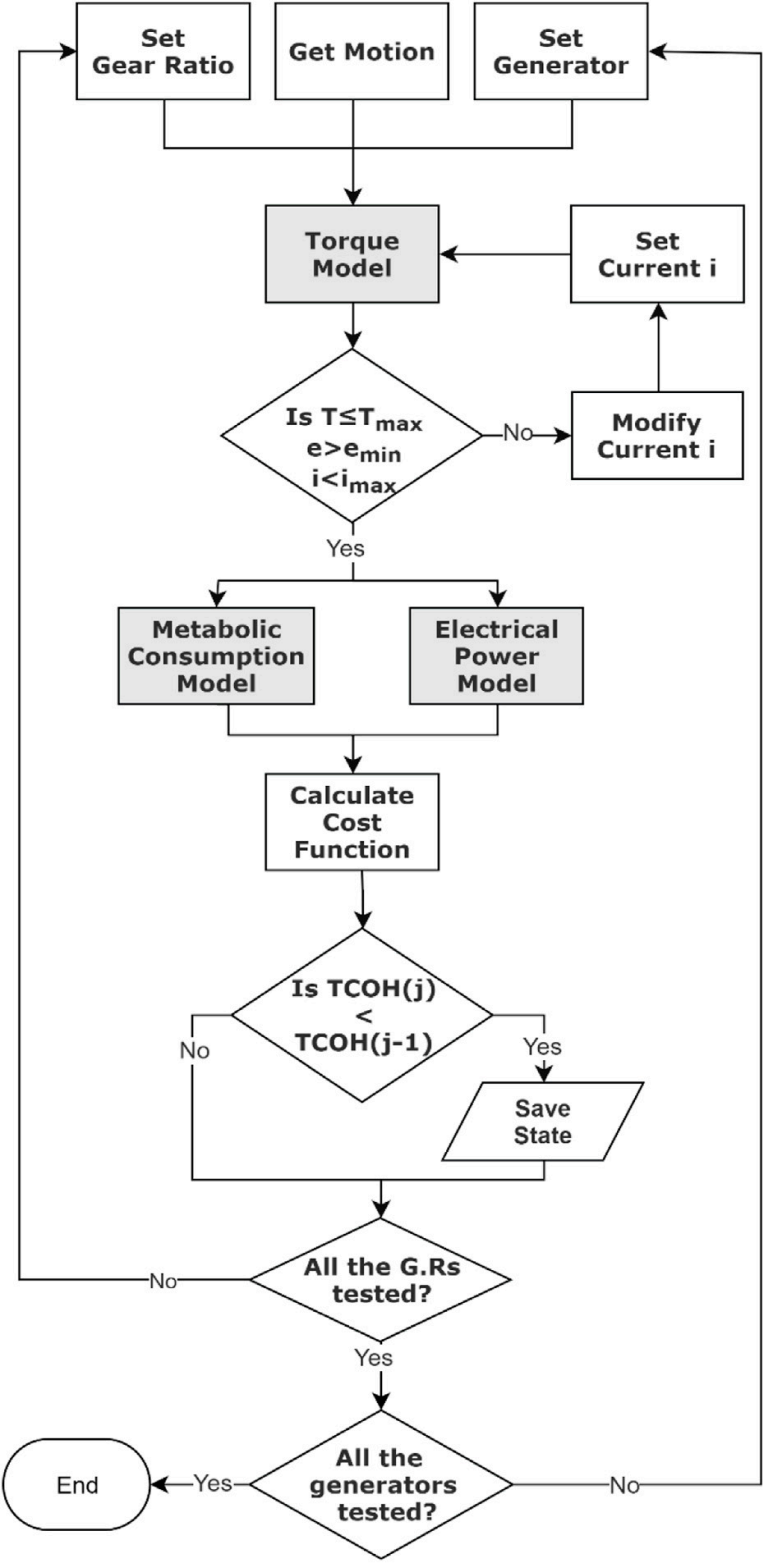
Block diagram of the optimization algorithm that ran through all the states and found the best one.

We applied our optimization for level walking where the input for motion is the knee joint average angular data. The timing of the harvesting algorithm was based on mapping the average knee joint angle profile to the average negative power profile. In a previous experiment, we found significant variation between subjects with regard to the start and end times of the phases where the knee was performing negative work (Shkedy Rabani et al., 2022). Thus, to avoid applying an incorrect torque profile during the gait (i.e., one that would lead to harvesting during positive power phases), the harvesting started at one standard deviation after our algorithm detected the start of the negative phase and ended at one standard deviation before the end of the negative phase. The optimization was solved using an in-house algorithm that is based on a grid search of the discretized parameter space. The optimization parameters are the electrical current through the generator (vector of 100 data points, where each point can be assigned a value from 0 to 10 A), the gear ratio (from 50 to 512 in steps of 1), and the motor (e.g., rotor inertia, torque constant, terminal resistance, nominal maximum current, speed constant, and mass). The algorithm was implemented in Matlab (MathWorks, Natick, MA, United States) and is represented in Figure 3.

To demonstrate our optimization algorithm, we tested 11 different DC brushless motors that, according to our engineering judgment as well as the experience of others (Donelan et al., 2008), (Shepertycky and Li, 2015), could be good candidates. Thus, we tested motors from (Donelan et al., 2008) and other motors that met the following criteria: mass of less than 250 g, electrical power ranging from 10 to 100 W, and voltage ranging from 10 to 60 V (our system could not function with a higher input voltage). This framework could be used by developers of other devices to test different motors and find the best motor for their application. For convenience, all motors were manufactured by Maxon (Sachseln, Switzerland). They differed in their mechanical and electrical characteristics, as shown in Table 1.

**TABLE 1:** Motors’ mechanical and electrical specifications.

Motor no.	Diameter [mm]	Width [mm]	MOI [gcm^2]	Weight [g]	Speed constant [rpm/v]	Torque constant [mNm/A]	Internal resistance [Ω]
323218	22	48.5	5.54	125	680	14	0.53
327739	22	48.5	5.54	125	145	66	13.50
323217	22	48.5	5.54	125	907	10.5	0.32
313320	40	26	10.5	170	565	16.9	0.52
339244	40	36	24.2	240	303	31.5	0.81
339243	40	36	24.2	240	572	16.7	0.37
339285	45	12.8	135	110	380	25.1	0.41
251601	45	12.8	135	110	285	33.5	0.98
339287	45	12.8	135	110	95	101	7.41
339241	40	26	10.5	170	1070	8.95	0.25
339286	45	12.8	135	110	201	47.5	2.77

#### 2.4.5 Building and testing the energy harvester

To evaluate our model, two exoskeletons, one for each leg, were built based on the optimization results (the list of the components and their masses is provided in Appendix B). Two experiments were then performed. The first was with a single male subject (1.8 m, 85 kg) walking with the device while a torque meter (mini 45, ATI, Apex, NC, United States) was installed at the knee joint (Appendix C).

**FIGURE 4:**
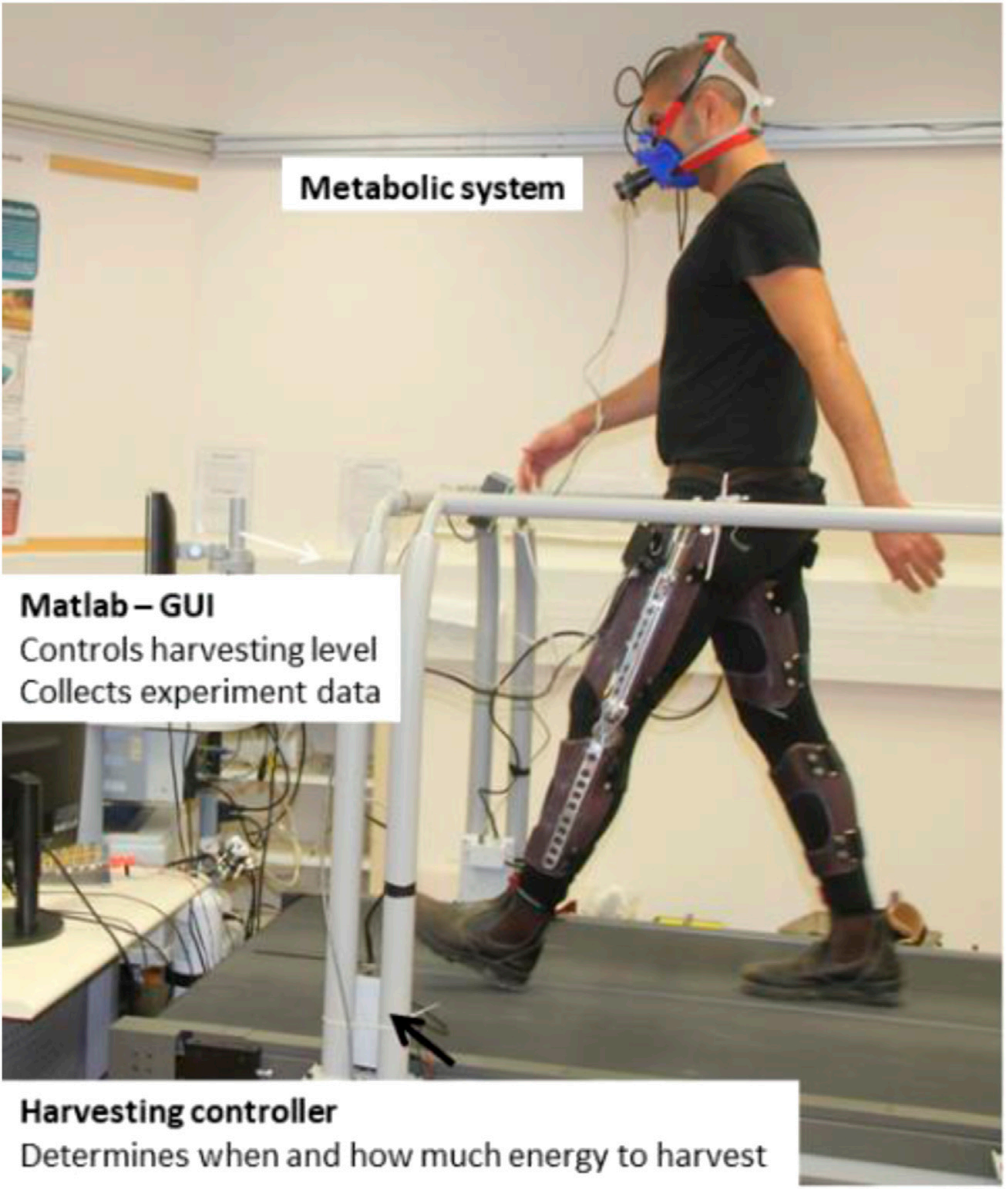
The experimental system included the Matlab graphical user interface (GUI), which sets the harvesting level and collects the experiment data; metabolic system; energy harvester for each leg; and harvesting controller for each leg, which determines when to harvest and how much (based on the harvesting level).

This experiment aimed to validate our modeling predictions (torque profile and energy harvested). In the next experiment, we studied the effect of different harvesting levels on the change in metabolic effort. Four male subjects (181.5 ± 2.6 cm, 85.3 ± 7.5 kg, 28–32 years) wore two harvesters, one on each leg, with each device controlled separately (the torque meter was removed to reduce device mass). They walked on a treadmill at a speed of 1.3 m/s under six different conditions: mechanically disconnected (wearing the device as a dead weight) and five harvesting states (15%, 22.5%, 30%, 37.5%, and 50%), each one representing a ratio of resistance torque applied to the knee joint based on normal gait data (Shkedy Rabani et al., 2022). Each condition was applied for a duration of 7 min of walking, where the last 3 min of the condition were used to calculate the metabolic rate. Between each condition, there was a 5-min resting period. The order of the harvesting states was randomized. The subjects completed the experiment twice; the first session was for training purposes, allowing the subjects to get used to the device, while the second session is the one reported here. The time interval between the two sessions ranged from 1 to 3 weeks. The harvester controller was an embedded device that used the PIC24FJ32GA102 microcontroller to control the torque of the generator and to run the detection algorithm (which determined the harvesting phases based on the real-time knee joint angle). The microcontroller consumed approximately 0.25 W for its operation. Since a specific load-oriented voltage control loop has not been realized, and to prevent overvoltage when unloaded, a dumping resistor with autonomous control was used. We also used this resistor to calculate the electrical power output (average of the last 3 min). Further information on the harvesting system can be found in (Cervera et al., 2016), (Rubinshtein et al., 2014). Metabolic rates were measured using the Quark system (Cosmed, Rome, Italy). Figure 4 shows the experimental setup.

#### 2.4.6 Statistical analysis

We first tested whether, for the group, there was an effect of condition (i.e., harvesting state) on electrical power and metabolic rate. Next, for each individual subject, we used their breath-to-breath data to determine whether there was an effect of condition on metabolic rate. In both cases, we used single-factor ANOVA with a post hoc Tukey Honest Significant Difference HSD test and a significance level of *p* less than 0.05.

## 3 Results and discussion

### 3.1 Optimization

**FIGURE 5:**
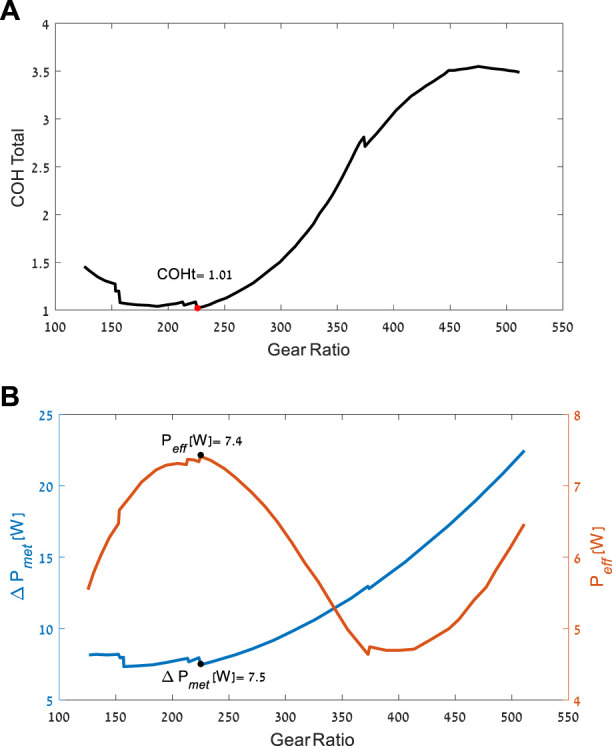
Optimization results for motor no. 323218. **(A)** The best possible TCOH as a function of gear ratio. **(B)** The best possible change in metabolic power, and the amount of electrical power produced, as a function of gear ratio.

The optimization process yielded the optimal gear ratio and torque profile to minimize the cost function (TCOH) for each of the motors. Figure 5 presents the results for motor no. 323218, which achieved the best TCOH. The comparison between the motors is presented in Table 2. Even though all the motors were considered good candidates for the device, there was approximately a six-fold difference in the value of the optimization cost function across the different models. The changes in gear and motor combination mass resulted in a difference of less than one metabolic W between the different combinations.

**TABLE 2:** Optimization results for each of the motors.

Generator No.	Gear ratio	TCOH	$P_{eff}$ [W]	$\Delta P_{met}$ [W]
**323218**	**225**	**1.01**	**7.40**	**7.50**
323217	210	1.09	7.16	7.84
327739	145	1.10	5.63	6.17
313320	140	1.26	7.09	8.91
339244	125	1.53	8.17	12.50
339241	192	1.88	6.16	11.57
339243	133	1.91	7.20	13.76
339285	227	3.47	36.78	127.69
251601	258	3.61	45.39	163.95
339286	186	4.40	19.79	87.00
339287	152	6.38	9.35	59.64

Generator no: the model number from Maxon’s catalog; $P_{eff}$ : the average electrical power per cycle; Δ*P*_*met*_: the average change in the metabolic power.

**FIGURE 6:**
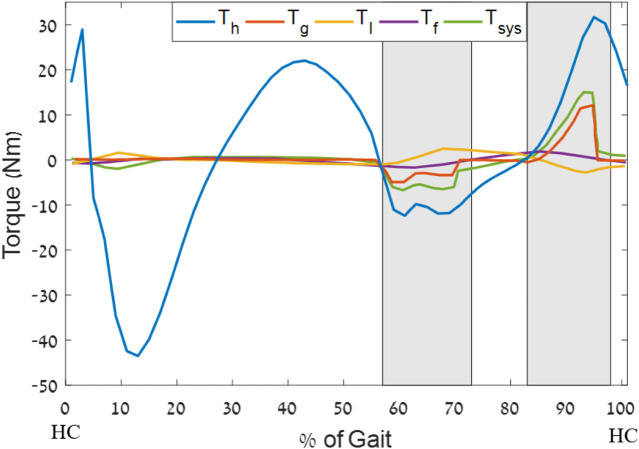
Optimal torque profile example for a 50% harvesting level (generator no. 323218), where $T_{h}$ is the standard human walking gait torque profile (Winter, 2009) scaled for an 83-kg human, $T_{g}$ is the optimal generator torque profile that is applied to the knee, $T_{I}$ is the torque felt by the user due to the moment of inertia, $T_{f}$ is the friction braking torque, and $T_{sys}$ is the total torque that the system applies to the knee. The shaded areas are the phases of energy harvesting. Note that we did not attempt to harvest for the entire durations of K3 and K4 due to variation in the population. The x-axis is normalized to the percentage of the gait cycle from heel contact (HC) to HC.

In the conceptual design used by the optimization, the generator is rigidly connected to the brace mechanism at all gait phases and causes mechanical torque resistance during the positive work phases (in these phases, more torque resistance will increase the metabolic effort). Thus, the optimization favors generators with low effective MOI (function of both the motor and the gear ratio), as this decreases the mechanical resistance during positive joint work phases, when the generator is not active (zero current; see Figure 6). Hence, the top seven generators had relatively low MOIs. The top three had the same motor design with different electrical properties. These results of preference for motors with a gear combination are in line with the changes that Shepertycky et al. (2021) made to improve their previous device (Shepertycky and Li, 2015) and the results from (Rubinshtein et al., 2012; Cervera et al., 2016). Further, it should be noted that the optimizations considered the user’s effort. Solutions such as higher gear ratios may produce more electricity, but they lower the conversion efficiency of the system, increasing the inertia and thus requiring more effort from users, especially in the non-harvesting phases of the gait. This was avoided by the optimization.

### 3.2 Model validation

**FIGURE 7:**
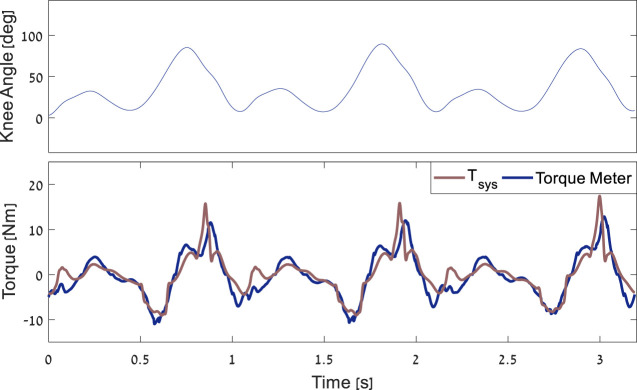
Example of the comparison between the torque meter results and the model over three gait cycles. $T_{sys}$ is the predicted torque that the system applies on the knee.

A harvesting device was built using motor no. 323218 (EC-4pole 22 Ø22 mm) and a gear ratio of 243:1, with a 3:1 bevel gear and 81:1 planetary gear. To validate the system torque model, an external torque meter was mounted on the harvester. The torque meter readings were then compared with the harvesting torque profiles from the model. The comparison was performed for several torque profile levels and, for each level, over approximately 15 cycles of the subject walking at 1.3 m/s. These experiments revealed a goodness of fit between the model and the torque meter of *R* ^2^ = 0.87 Figure 7. Further, two calculations were performed for the total efficiency of the device: 1) Calculated based on specifications of the two gears (Apex 85%, Bevel 95%) of the electrical harvesting system 88% (Cervera et al., 2016). Thus, this calculation results in approximately 71% efficiency. 2) Using the torque meter to calculate mechanical energy input, the electrical power-generated efficiency was calculated to be approximately 65%.

### 3.3 Experimental results

**FIGURE 8:**
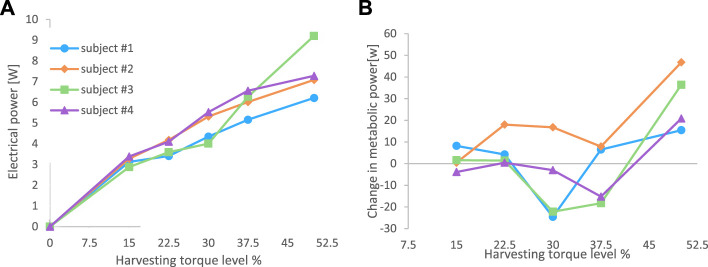
Results from the experiments on four subjects. **(A)** Electrical power output of the system for each of the subjects at six harvesting levels. **(B)** Metabolic change relative to walking with the device disconnected (dead weight). The figure data is in Appendix D.

For all the subjects, an increase in the harvesting torque level resulted in an increase in electrical power output (Figure 8A), where the ANOVA showed that there was a significant difference in electrical power output between conditions (*p* < 0.05), and post hoc Tukey HSD tests revealed that there were significant differences only between non-neighboring conditions. For example, the 22.5% harvesting level was not significantly different from the 15% or the 30% levels, but it was different from the 37.5% and 50% levels. The metabolic change relative to walking with the device disconnected was calculated for each of the subjects at each of the harvesting levels (Figure 8B). The ANOVA results revealed that overall (i.e., across all subjects), the effect of the harvesting level on the metabolic change was significant, and the post hoc tests revealed that the 30% level was significantly different from the 50% condition. When testing the metabolic rate at the different experimental conditions for each of the subjects, the following results were obtained. For Subject 1, the 30% harvesting level (corresponding to the lowest value of metabolic change) was significantly different from all the other conditions. For Subject 2, the 15% condition was statistically different from the 22.5%, 30%, and 50% conditions but not from the 37.5% condition. For Subject 3, the 30% condition was different from all other conditions except 37.5%. For Subject 4, all the conditions were different from the 50% condition. Note that negative values in Figure 8B indicate that electrical energy was generated while the metabolic power (effort) was reduced (compared to the disconnected condition). These results suggest that the harvesting level of the device should be subject specific. This is in line with the findings of, which showed that different levels of harvesting resulted in different changes in metabolic power values for different individuals (Donelan et al., 2008), (Shepertycky and Li, 2015)]. As well as with the findings of, which showed that individual optimization of the actuation using the human-in-the-loop method could improve the performance of exoskeletons relative to a generic mode (Zhang et al., 2017), (Lee et al., 2018). Thus, future harvesters should customize their final torque profile using this method. Further, many exoskeletons that attach to the body are custom-made for each subject [e.g., 16], which improves the energy transfer between the exoskeleton and enhances users’ comfort.

**FIGURE 9:**
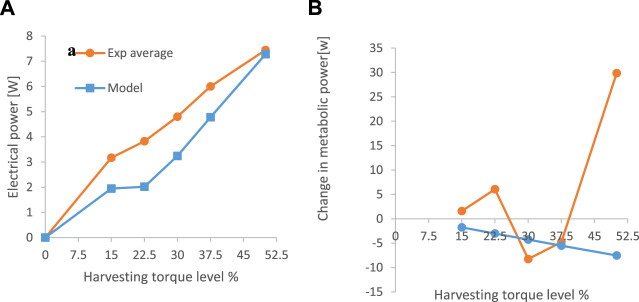
Comparison between the average results from the experiment and the predictions of the simulation. **(A)** electrical power harvested. **(B)** metabolic power.

Comparison of the model prediction and the average experimental results (Figure 9A) revealed that the harvesting energy was similar in both cases, albeit with slightly higher values for the experimental results at low harvesting levels. This could be explained by the fact that the device managed to harvest at a lower voltage than the limit in the simulation. Regarding the metabolic differences between the harvesting and mechanically disconnected conditions (Figure 9B), while the model predicted a continuous reduction in metabolic power with an increase in the harvesting level, the experimental results showed a complex shape with a minimum at 30% and a large increase in metabolic power at 50%.

### 3.4 Limitations and future work

In this study, we used a model based on the work of Margaria et al. (1968), which relates mechanical work performed by the body to metabolic rate. This model was evaluated using experimental data of walking on sloped and level ground and was found to be a good fit (R2 > 0.9) compared to more complex models that include muscle (Koelewijn et al., 2019). However, in our experiments, as was also observed by Collins et al. (2015), at some point there was an increase in metabolic rate as the device provided more assistance at the joint level. We believe that this could be due to several mechanisms. First, recent work shows that even with more complex models with muscle (Umberger et al., 2003) the simulation failed to find the same optimal assistance torque that was found in the experiments in (Franks et al., 2020). Second, as was also the case in previous studies (Uchida et al., 2016), (Dembia et al., 2017), in our optimization there is an assumption that the walking kinematics and kinetics do not change with the load. Yet, while this holds true for lower loads, for higher loads, there appears to be an effect of the load on the walking patterns at all joints (Donelan et al., 2008), (Shepertycky and Li, 2015). An alternative to the above method might be to use the experimental results to create a function that relates the level of harvesting to the change in metabolic power and to use this relation during the optimization for the design of the next device.

Regarding future work, Shepertycky et al. (2021) recently built and tested a device that reduced metabolic effort compared to walking without a device while producing 0.25 W of electricity from the late swing motion. The current device was unable to reduce the overall metabolic effort (i.e., walking with no device compared with walking with the activated device). However, the design (with the motor connected with no clutch mechanism) enabled harvesting energy during both flexion and extension. This enables more potential energy to be harvested, for example, in our 30% condition, in which the best for most of the subject. The device harvested an average electrical power of 4.8 W while reducing the metabolic power by 8.26 W compared to walking with the harvester as a dead weight. Using the framework proposed by Schertzer and Riemer et al. (2015), we found that in theory, a harvester with a mass of less than 0.5 kg could produce approximately 5 W, which is 40 times more than the best result achieved in (Shepertycky et al., 2021) and would reduce the metabolic effort. It should be noted that our current energy harvester is a prototype that was designed to test a concept. When using the device in these pilot experiments, we were able to generate an average of 12 W during walking at a torque level of approximately 80%. Thus, a design for 30% of this torque level could be achieved using a smaller motor and a lighter structure, as the force and torque would be smaller. A further reduction in the device’s mass could be achieved by using a material such as carbon fiber, which has a tensile strength-to-mass ratio that is between two and four times higher than the aluminum 7075 T6 that we employed. This means that a structure that could hold the same load would be two to four times lighter.

This study tested a knee energy-harvesting device that could generate electricity during the joint’s mechanical negative work phases of the gait, both at flexion and extension. Thus, it could harvest in K1, K3, and K4, as opposed to previous harvesters, which could only harvest during the late swing (K4, Figure 2). Our optimization preferred generators with effective low MOI. This is advantageous for designing with direct drive, as a one-way roller clutch with high MOI (Donelan et al., 2008; Shepertycky and Li, 2015) that might result in disengagement of the generator towards the end of the late swing phase. This disengagement is due to large kinetic energy stored that electrical harvesting needs to remove to slow down the angular velocity of the generator, at a rate needed to follow the angular velocity provided to the generator by the knee (Rubinshtein et al., 2012). Further, the highly effective MOI means that the system is designed for one specific gait and will not work as well in other conditions (e.g., slower walking steps or running). Thus, the direct-drive approach with low MOI should enable harvesting more energy and other conditions with more negative joint work than level walking (e.g., running) (Riemer et al., 2021). A recent development in exoskeletons is a device with regenerative braking (Zhu et al., 2019), which can harvest and return energy and provide assistance when the joint is performing either positive or negative work. Therefore, our results might support the effort to build exoskeletons that have a minimal need for external power yet can change their assistive torque profiles as needed.

## 4 Conclusion

In this study, we presented an optimization method for the design of an energy harvester system during level walking. This method enabled testing many different motor and gear combinations and could be used by developers of other devices to achieve the best design for their application. Further, our device enabled us to harvest energy from all gait phases and not only from late swing phase. Our experiments revealed that for all the subjects, there was at least one harvesting condition where the metabolic power required for generating electrical energy was close to zero or less than zero. These results suggest that a lighter, similar design might be able to improve user metabolic performance in the future.

## Figures and Tables

**FIGURE 10:**
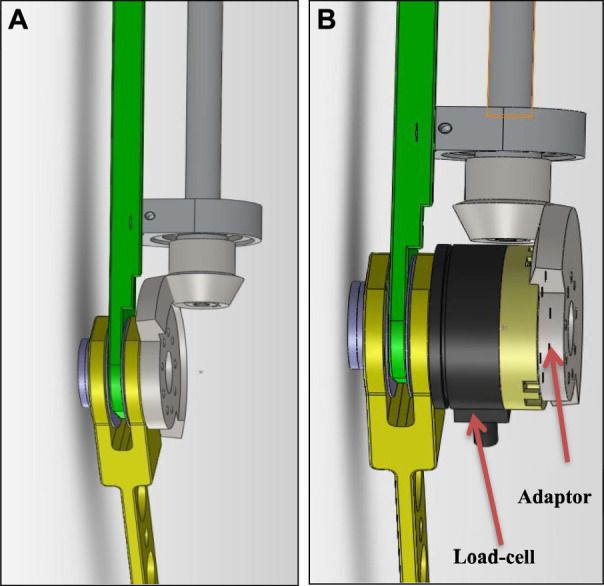
Illustration of the method of mounting the torque meter. **(A)** Normal use without the torque meter. **(B)** Configuration with the torque meter attached.

## Data Availability

The raw data supporting the conclusion of this article will be made available by the authors, without undue reservation.

## Appendix A: BLDC- DC conversion

The following set of equations defines the DC equivalent circuit constants of the generator:

$$K_{e}=\frac{1}{K_{n}}=\frac{1}{\sqrt{3}{\tilde{K}}_{n}}$$
(22)

where ${\tilde{K}}_{n}$ is the data sheet EMF constant, and $K_{e}$ is the equivalent EMF constant;

$$K_{m}=\frac{1}{3}{\tilde{K}}_{m}$$
(23)

where ${\tilde{K}}_{m}$ is the data sheet momentum constant, and $K_{m}$ is the equivalent torque constant; and

$$R_{i}=\left(\frac{\sqrt{3}}{2}{\tilde{R}}_{p}\right)\frac{2}{3}=\frac{1}{\sqrt{3}}{\tilde{R}}_{p}$$
(24)

where ${\tilde{R}}_{p}$ is the data sheet phase-to-phase resistance constant, and $R_{i}$ is the equivalent internal resistance.

The external load is bound to this equivalent circuit as well, and the transformation is given by

$$R_{l}=\frac{2}{3}{\tilde{R}}_{l}$$
(25)

where ${\tilde{R}}_{l}$ is the external load resistance in the three-phase circuit, and $R_{l}$ is the equivalent external load resistance.

## Appendix B: Masses of the device components

**Table:**

Part name	Weight per unit [g]	Quantity
Apex gearbox	265.83	1
MAXON 323218	125	1
K1300 45T	112.29	1
Knee axis house low	86.56	1
Knee axis house high	91.15	1
Main axis	36.26	1
K1300 15T	38.28	1
Bevel sensor adaptor	33.12	1
Big bearing house	16.04	1
Small bearing house	6	1
Gear holder front	4.69	1
Motor holder front	0	1
Motor holder back	3.96	1
7200-BE bearing	3	1
618–5 bearing	1.2	2
618–6 bearing	2	1
Knee hinge	1.16	1
Cover 2	50.8	1
Orthopedic parts	700	1
Total weight per leg	1545.42	

## Appendix C: Mounting the torque meter

In order to verify the system’s torque model, we needed to be able to measure the torque that the system applied to the knee during a walking gait while the energy harvester was harvesting energy. The torque meter is a heavy component and therefore needed to be detachable so that it could be removed once the model validation was completed. The torque meter used in the study was the ATI mini 45, which is a 6DOF load cell. To mount the torque meter, we designed an adaptor that we mounted on the torque meter so that the bevel gear could be connected to it (Figure 10).

## Appendix D: Experiment data in table form

**TABLE D1:** Metabolic power differences (Harvesting-Disconnected); bold indicates the lowest number for each subject.

Harvesting level %	15.0	22.5	30.0	37.5	50.0
Sub#1	8.19	4.25	**−24.59**	6.49	15.43
Sub#2	**0.45**	18.05	16.79	7.88	46.75
Sub#3	1.55	1.40	**−22.20**	**−**18.31	36.40
Sub#4	**−**3.85	0.45	-3.04	**−15.22**	20.82
Average	1.59	6.04	**−**8.26	**−**4.79	29.85

**TABLE D2:** Electrical power produced at each harvesting level.

Harvesting level %	0	15	22.5	30	37.5	50
Sub #1	0	3.13	3.41	4.35	5.16	6.21
Sub #2	0	3.28	4.18	5.32	6.02	7.08
Sub #3	0	2.88	3.59	4.01	6.27	9.21
Sub #4	0	3.38	4.10	5.53	6.56	7.28
Average	0	3.17	3.82	4.80	6.00	7.45
